# Cell-type-specific modulation of neocortical activity by basal forebrain input

**DOI:** 10.3389/fnsys.2012.00079

**Published:** 2013-01-09

**Authors:** Henry J. Alitto, Yang Dan

**Affiliations:** Division of Neurobiology, Department of Molecular and Cell Biology, Helen Wills Neuroscience Institute, Howard Hughes Medical Institute, University of CaliforniaBerkeley, CA, USA

**Keywords:** acetylcholine, visual cortex, vasoactive intestinal peptide, parvalbumin, layer 1

## Abstract

Activation of the cholinergic neurons in the basal forebrain (BF) desynchronizes cortical activity and enhances sensory processing during arousal and attention. How the cholinergic input modulates the activity of different subtypes of cortical neurons remains unclear. Using *in vivo* two-photon calcium imaging of neurons in layers 1 and 2/3 of mouse visual cortex, we show that electrical stimulation of the BF bi-directionally modulates the activity of excitatory neurons as well as several subtypes of inhibitory interneurons. While glutamatergic activity contributed to the activation of both excitatory and inhibitory neurons, the contribution of acetylcholine (ACh) was more complex. Excitatory and parvalbumin-positive (PV+) neurons were activated through muscarinic ACh receptors (mAChRs) at low levels of cortical desynchronization and suppressed through nicotinic ACh receptors (nAChRs) when cortical desynchronization was strong. In contrast, vasoactive intestinal peptide-positive (VIP+) and layer 1 interneurons were preferentially activated through nAChRs during strong cortical desynchronization. Thus, cholinergic input from the BF causes a significant shift in the relative activity levels of different subtypes of cortical neurons at increasing levels of cortical desynchronization.

## Introduction

The cholinergic input from the BF to the neocortex has been implicated in a variety of cognitive functions, including memory, attention, and sensory processing (Everitt and Robbins, 1997; Kilgard and Merzenich, 1998; Hasselmo and Sarter, 2011; Newman et al., 2012). The BF cholinergic neurons show high firing rates during alert wakefulness and rapid-eye-movement (REM) sleep, both of which are associated with low-amplitude, high-frequency (desynchronized) electroencephalogram (EEG). In contrast, during quiet wakefulness and slow-wave sleep with synchronized EEG, these cholinergic neurons are much less active (Jones, 2004; Lee et al., 2005). Electrical stimulation of the BF triggers cortical desynchronization (Metherate et al., 1992) and improves sensory processing (Goard and Dan, 2009), indicating a causal relationship between BF activation and wakeful/alert brain states.

Although the influence of the BF cholinergic input on cortical population dynamics has been well characterized, the effects on specific cell types remain poorly understood, especially in an intact network *in vivo*. The BF provides the only long-range cholinergic input to the neocortex (together with glutamatergic, GABAergic inputs), projecting to all six layers (Lehmann et al., 1980; Mechawar et al., 2000; Henny and Jones, 2008; Hassani et al., 2009). Both excitatory and inhibitory cortical neurons express ACh receptors, but the ionotropic nAChRs and the metabotropic mAChRs are known to be differentially expressed among different cell types (Kawaguchi, 1997; Porter et al., 1999; Freund, 2003; Markram et al., 2004; Lawrence, 2008). Among the GABAergic interneurons, there is a high degree of diversity in their molecular markers, electrophysiological properties, and innervations patterns (Markram et al., 2004; Ascoli et al., 2008; Xu et al., 2010; Kubota et al., 2011). Recent studies have shown that nearly all cortical GABAergic interneurons can be classified into three non-overlapping populations with distinct molecular markers: PV, somatostatin (SOM), and the ionotropic serotonin receptor 5HT3aR (Lee et al., 2010; Rudy et al., 2011). The 5HT3aR+ population, including all the VIP+ and layer 1 interneurons (Christophe et al., 2002; Rudy et al., 2011), can be activated by nAChR agonists (Porter et al., 1999; Christophe et al., 2002; Lee et al., 2010), while the membrane potential of pyramidal neurons and SOM+ and fast-spiking interneurons can be modulated through mAChRs (McCormick, 1992; Xiang et al., 1998; Fanselow et al., 2008). In addition to the cholinergic input, GABAergic axons from the BF make synaptic contacts with excitatory neurons as well as inhibitory interneurons, while glutamatergic axons selectively target non-PV+ interneurons (Henny and Jones, 2008). Thus, activation of the BF input is likely to exert diverse effects on different types of cortical neurons, and characterization of these effects is crucial for understanding how the BF input modulates cortical function at the microcircuit level.

In this study, we used two-photon calcium imaging to measure the effects of BF activation on cortical excitatory neurons and several subtypes of inhibitory interneurons, each labeled with a fluorescent marker in a transgenic mouse line. We found significant BF modulation of all the cell types examined. Pharmacological experiments showed that while mAChRs and glutamate receptors contributed to the excitation of all layer 2/3 cell types, the effect of nAChRs is more complex. It caused strong activation of VIP+ and layer 1 interneurons but suppression of excitatory neurons and PV+ interneurons, presumably through the inhibitory connections from the VIP+ and/or layer 1 neurons to excitatory and PV+ neurons. Furthermore, the contribution of nAChRs increased sharply with the level of cortical desynchronization, suggesting that strong cholinergic input causes not only a change in the cortical network dynamics but also a shift in the primary source of cortical inhibition from the PV+ neurons to the VIP+ and layer 1 interneurons.

## Materials and methods

### Surgery

All experimental procedures were conducted according to the rules and regulations set forth by the Animal Care and Use Committee at the University of California, Berkeley. Adult male and female transgenic mice (post-natal days 60–180) were anesthetized with urethane (intraperitoneal, 1.3 g per kg of body weight). Mice were restrained in a stereotaxic apparatus (David Kopf Instruments) and their body temperature was maintained at 37.5°C via a heating pad. Cortical EEG was recorded (Model 1700 Amplifier, A-M Systems) through bone screws inserted into the skull rostral to bregma and analyzed with a custom Matlab program. Bipolar stimulating electrodes were stereotaxically implanted in the left nucleus basalis (AP = −0.5 mm, ML = 1.7 mm, DV = 4.0–5.0 mm). The position of the electrode was adjusted until electrical stimulation (50 × 0.1 ms pulses at 100 Hz) successfully desynchronized cortical EEG (decreased EEG power at 1–10 Hz). The electrode and a custom metal head plate were then cemented to the skull and attached to stabilization posts. A ~1.5 mm craniotomy was made over primary visual cortex (ipsilateral to the stimulation electrode) and 1.5% agar was applied to the cortical surface to provide additional stability and prevent dehydration of the cortical tissue. Visual cortex was labeled with calcium indicator dye [1 mM Oregon Green 488 BAPTA-1 AM (OGB-1, Invitrogen), 0.2 mMAlexaFluor 594 hydrazide (Invitrogen), 10% dimethylsulphoxide (DMSO, Invitrogen), 2% (wt/vol) Pluronic F-127 in HEPES-buffered saline] via visually guided bolus loading (Garaschuk et al., 2006). Imaging experiments began ~60 min after the dye injection. In a subset of experiments 0.1 mM SR-101 (Invitrogen) replaced AlexFluor 594 hydrazide to label cortical astrocytes (Nimmerjahn et al., 2004).

### Two-photon imaging

The custom-made two-photon microscope (Tsai et al., 2002) was controlled using custom software. The intensity of the excitation from a tunable femtosecond laser (Wideband, Tsunami Mode-Locked Ti: Sapphire Laser, Spectra-Physics) was controlled by a Pockel cell (302 Driver; 350-50 modulator, Conoptics). The excitation laser was focused using a 40X/0.8 NA infrared objective (LUMPLFLN, Olympus). Fluorescence was collected after a dichroic mirror (650dcxr, Chroma) and an emission filter (Chroma: 540-40/2P). Emission light was then divided into green (>580 nm) and red channels (<580 nm) by a second dichroic mirror (580dcxr, Chroma) and collected by photomultiplier tubes (R3896, Hamamatsu). Frames of 256 × 256 pixels were acquired at ~2 Hz using bi-directional scanning. Different combinations of excitation and emission wavelengths were used to identify fluorescence of OGB-1 (λex = 800 nm, λem = 420–580 nm), Alexa 594 (λex = 800 nm, λem = 580–650 nm), GFP (λex = 920 nm, λem = 420–580 nm) and tdTomato (λex = 920 nm, λem = 580–650 nm). Images were corrected for horizontal motion artifact using the TurboReg plug-in for ImageJ (NIH) and imported into Matlab for quantitative analysis.

Excitatory neurons and several types of GABAergic neurons were targeted in the current study. To visualize CaMKIIα+ excitatory neurons, PV+ interneurons, and VIP+ interneurons we crossed the corresponding cre-dependent mouse line (Tsien et al., 1996; Taniguchi et al., 2011) with a loxP-flanked tdTomato reporter mouse (Jackson Labs). GFP labeled SOM+ interneurons were imaged in homozygous GIN mice (Jackson Labs) (Oliva et al., 2000) and heterozygous GIN mice (GIN mice × C57BL/6 wild-type mice). CaMKIIα-cre × tdTomato mice were also used to identify layer 1 inhibitory neurons, defined as unlabeled cells (negative for tdTomato, negative for SR-101) near the pial surface (<90 μm) where the cell density was low relative to layer 2/3 and excitatory neurons were absent. SR-101 was not used when excitatory neurons (or other tdTomato labeled neurons) were the targeted cell type. Visually approximated cortical depth for imaging varied between ~20–90 μm for layer 1 and ~100–300 μm for layer 2/3 neurons and astrocytes.

Neurons were imaged during BF stimulation (minimum 10 stimulation events). For each cell, a region of interest (ROI) surrounding the cell body was manually selected using a custom MATLAB program. Pixel values were summed within the ROI for each frame. The resulting time-lapse fluorescence was converted to a fractional change in fluorescence, dF/F(t) = [F(t)–Fo(t)]/Fo(t) where Fo(t) is s the mean of the fluorescence over the course of the imaging session (~6–10 min). A cell's response was classified as positive or negative if it exceeded ±4 × SD of the pre-stimulation baseline (0–2 s before stimulus onset). If the response could be classified as both negative and positive (uncommon for all cell types except PV+ interneurons) it was characterized by the event that occurred with the shorter latency. If the inter-stimulation interval was less than 15 s (24% of cells), the period prior to the onset of the entire train of stimulation was used as the pre-stimulation baseline.

### Pharmacology

For pharmacology experiments, genetically labeled neurons were identified and their positions relative to other cells (labeled and unlabeled), vasculature, and the pial surface were mapped. Each drug was topically applied to the cortical surface (~45–60 min) to block mAChRs (1 mM atropine), nAChRs (3 mM mecamylamine), or ionotropic, glutamatergic receptors (AMPARs, 4 mM CNQX). After drug application the exact neurons imaged in the pre-drug experiments were located and imaged again in the “post-drug” stimulation experiment (same stimulation parameters as the pre-drug condition). Only cells that had statistically significant responses to BF stimulation (negative or positive) before and/or after drug application were included in the experimental data set. Pre- and post-drug response levels are reported as the peak response amplitude (dF/F) evoked by BF stimulation (0–5 s post-stimulation). For all pharmacology data similar results were found when analyzing response magnitude (sum of all contiguous bins with the same sign as the peak bin). Nonparametric sign tests (Matlab function signtest) were used to test for significant differences between the pre- and post-drug data.

### EEG correlation analysis

To determine the relationship between cortical desynchronization and the response levels of specific neuronal subsets we plotted the response magnitude as a function of the desynchronization index (1 – EEG power Post-Stim_1–10Hz_/EEGpower Pre-Stim_1–10Hz_). Neuronal responses (dF/F) were averaged for each individual experiment (all responsive cells imaged simultaneously).

## Results

**Figure 1:**
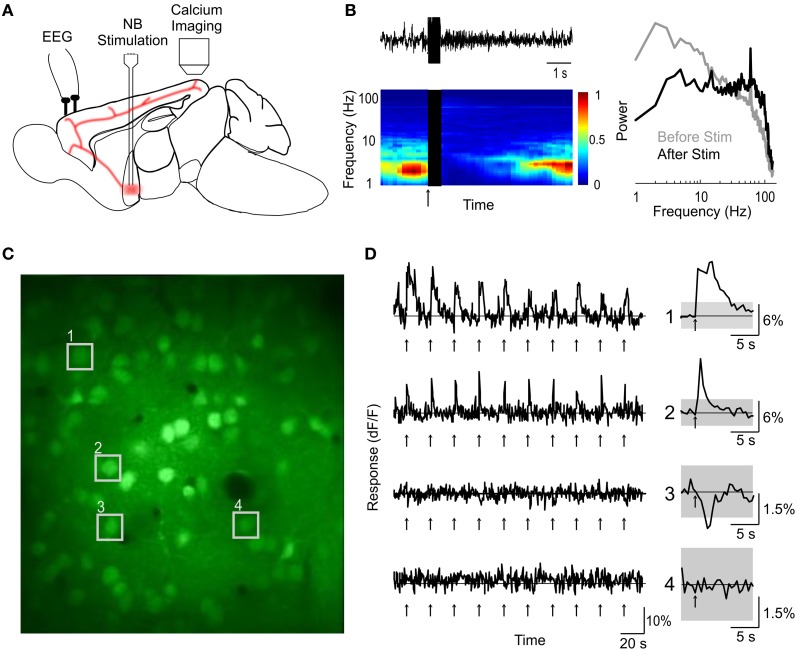
**Two-photon calcium imaging of basal forebrain modulation of cortical activity. (A)** Schematic illustration of experimental design. **(B)** An example of cortical desynchronization induced by BF stimulation. Top left, EEG trace of a single trial. Bottom left, EEG spectrogram averaged from 10 trials; blue, low amplitude; red, high amplitude; black, period of BF stimulation; arrow, stimulus onset. Right, amplitude spectra during a 2 s period pre-(gray) and post-(black) stimulation, averaged from 10 stimulation trials. **(C)** An example fluorescence image of visual cortex loaded with OGB-1 (depth, 210 μm). **(D)** dF/F traces of four example cells (indicated by numbers in **C**) during a block of 10 trials of BF stimulation (arrows). Right, average response over the 10 trials. Gray shading, 4× SD of baseline. Cells 1 and 2 responded to BF stimulation with significant increases in calcium level, cell 3 showed a significant decrease, and cell 4 was not significantly modulated.

We labeled cells in the primary visual cortex (V1) of urethane-anaesthetized mouse with the calcium indicator dye Oregon Green BAPTA-1 AM (OGB-1) using bolus loading (Garaschuk et al., 2006) (see “Materials and Methods”). The BF was stimulated with a bipolar electrode (75–150 μA, 500 ms, 100 Hz, repeated once every 5–30 s) while the calcium signals of individual V1 cells were monitored using two-photon imaging (Figure 1A). Successful activation of the BF cholinergic input, assessed by desynchronization of cortical EEG (Figure 1B), caused transient changes in intracellular calcium in a subset of the cells in layers 1 and 2/3 (Figures 1C,D). In the experiments with clear BF-induced EEG desynchronization (desynchronization index >0.3, see “Materials and Methods”), 9.4 ± 0.01% (SEM) of the cells in layer 2/3 showed a significant increase in calcium concentration (amplitude of dF/F >4 × SD of the baseline measured before stimulation), and 3.5 ± 0.03% showed a significant decrease (< −4 × SD). In contrast, when BF stimulation failed to induce clear EEG desynchronization (desynchronization index <0.3, *n* = 16), we observed little change in single cell calcium concentration (1.1 ± 0.04% significant increase, 0.2 ± 0.1% significant decrease). This suggests that the calcium responses are directly related to activation of the cholinergic input to the cortex.

### Cell-type specificity of response to BF stimulation

To determine whether the small fraction of cells responsive to BF stimulation belong to specific cell types, we performed BF stimulation in four transgenic mouse lines, each expressing a fluorescent marker in a specific cell type: CaMKIIα+ excitatory neurons and PV+, VIP+, and SOM+ inhibitory interneurons. In addition, astrocytes were labeled with SR101, and layer 1 neurons were identified based on their proximity to the pia surface and the lower cell density compared to layer 2/3.

**Figure 2:**
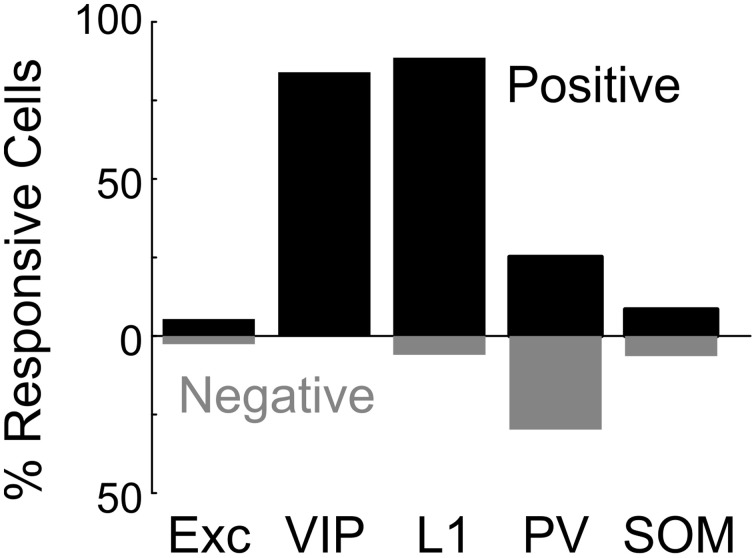
**Basal forebrain modulation of excitatory and inhibitory cortical neurons.** Shown is the percentage of significantly responsive neurons for each cell type. Black bar, significant positive response (dF/F > 4 × SD of baseline). Gray bar, significant negative response (dF/F < –4 × SD of baseline).

**Figure 3:**
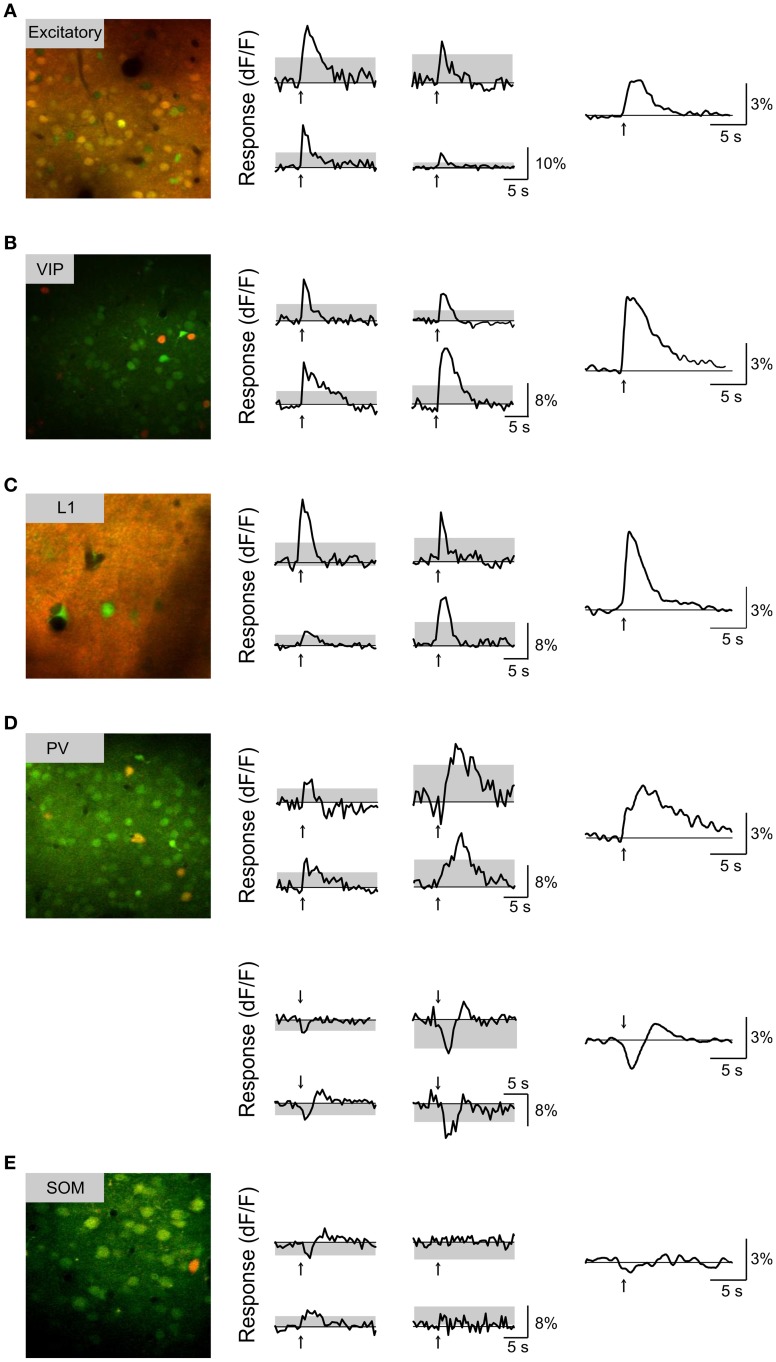
**Basal forebrain modulation of each subtype of cortical neurons. (A)** Left, Example fluorescence image from a CaMKIIα+ transgenic mouse (red, tdTomato; green, OGB-1). Middle, responses to BF stimulation (averaged from 10 trials) for four example neurons; arrow, stimulus onset; gray area, 4 × SD of baseline. Right, response averaged from all significantly responsive excitatory neurons (*n* = 71 positive dF/F responses, 34 negative dF/F responses). **(B)** Similar to **(A)**, for VIP+ neurons (*n* = 50 positive). **(C)** Layer 1 neurons (*n* = 30 positive, 2 negative). **(D)** PV+ neurons with positive (top panel, *n* = 39) and negative (bottom panel, *n* = 33) responses. **(E)** SOM+ neurons (red, GFP; green, OGB-1; *n* = 47, 4 positive, 3 negative).

**Figure 4:**
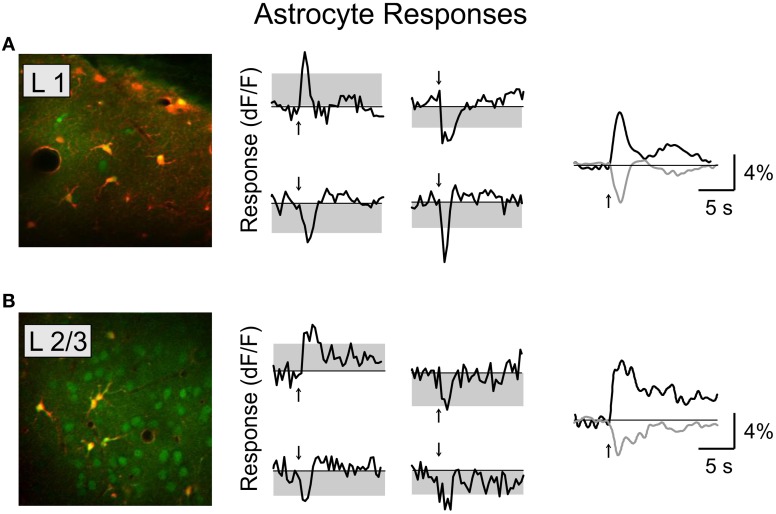
**Basal forebrain modulation of cortical astrocytes.** Left, astrocytes labeled with SR-101 in layer 1 **(A)** and layer 2/3 **(B)**. Red, SR-101; green, OBG-1. Middle column, four example responsive astrocytes from each layer. Right, average response for all the significantly responsive astrocytes in each layer (black, positive; gray, negative).

We found that BF stimulation evoked significant responses in subsets of all the cell types examined (Figures 2, 3). However, there were important differences across cell types. In the CaMKIIα+ mice (Figure 3A), only a small fraction of layer 2/3 excitatory neurons) showed significant positive (5.4%, increase in dF/F) or negative (2.6%) responses, much lower than the remaining unlabeled cells (25.5% positive, 7.3% negative). Among the inhibitory interneurons, most of VIP+ (Figure 3B, 84.2%) and layer 1 (Figure 3C, 88.6%) interneurons showed strong positive responses, while PV+ interneurons showed both positive (Figure 3D top panel, 25.2%) and negative (bottom panel, 29.8%) responses. Very few SOM+ neurons showed significant responses (Figure 3E, 8.5% positive, 6.4% negative), and their amplitudes were quite low, thus they were not further examined. The SR101 labeled astrocytes, on the other hand, showed more negative (Figure 4; 41% in layer 1, 25.5% in layer 2/3) than positive (15% in layer 1, 6.6% in layer 2/3) responses.

### Dependence on cholinergic transmission

**Figure 5:**
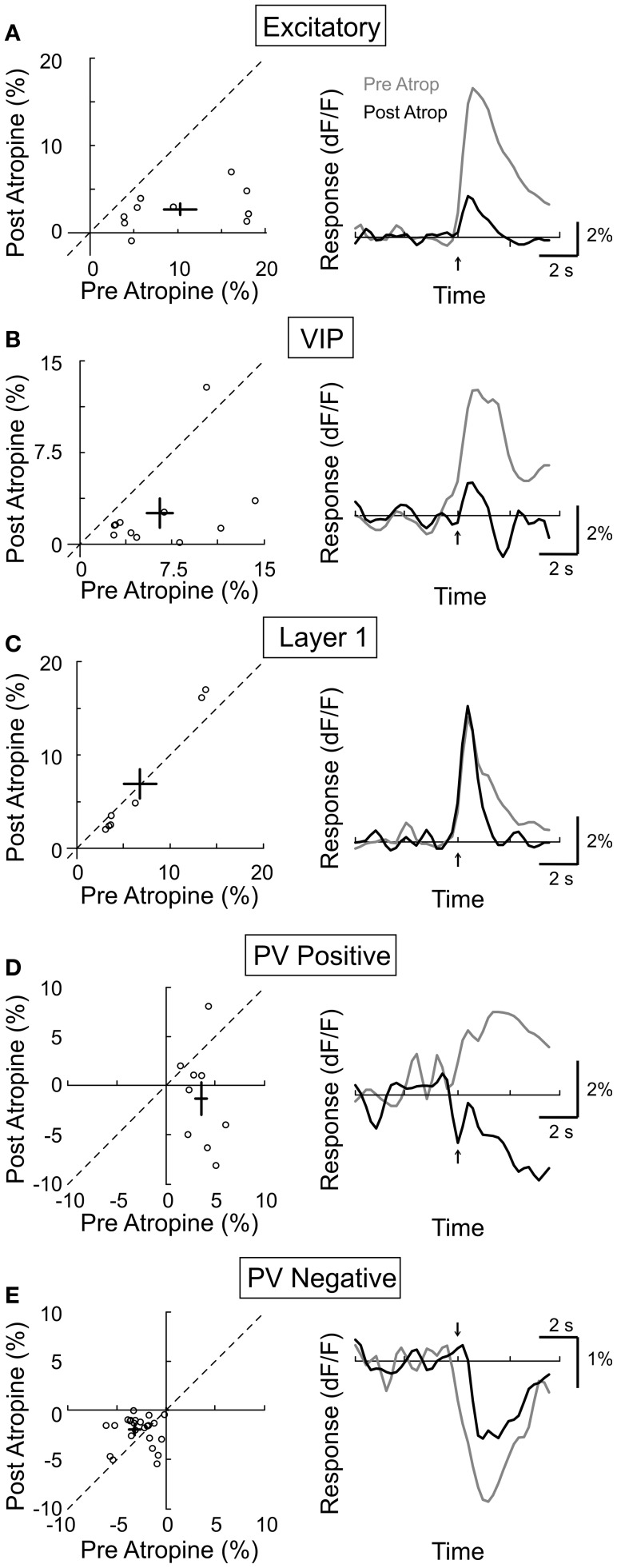
**Effect of atropine on BF modulation of cortical neurons.** Left column, post-atropine vs. pre-atropine response amplitude (dF/F). Each symbol, one cell; error bar, ±SEM. Right column, average response from all responsive cells in each subtype; gray, pre-atropine; black, post-atropine. **(A)** Excitatory neurons, decreased by 77% post-atropine (*p* < 0.0001, *n* = 10). **(B)** VIP+, decreased by 62% (*p* < 0.01, *n* = 10). **(C)** Layer 1, no significant change (*p* = 0.45, *n* = 6). **(D)** PV+ with positive responses, decreased by 136% (*p* < 0.05, *n* = 9). **(E)** PV+ with negative responses, decreased by 38% (*p* < 0.05, *n* = 26).

While electrical stimulation of the BF activates cholinergic as well as glutamatergic and GABAergic axons, we assessed the contribution of the cholinergic input by measuring the effects of mAChR and nAChR antagonists. Muscarinic AChRs are found in both excitatory and inhibitory neurons throughout all layers of the neocortex, and their activation is required for cortical desynchronization (Metherate et al., 1992; Erisir et al., 2001; Volpicelli and Levey, 2004). To test the contribution of mAChRs in the responses of individual neurons, we compared their calcium responses to BF stimulation before and after topical application of atropine (1 mM), a selective mAChR antagonist. We found that atropine application caused a strong reduction in the response amplitude of the excitatory neurons (Figure 5A, amplitude_pre_ = 10.3 ± 2.0, amplitude_post_ = 2.8 ± 0.6, *p* < 0.0001, *n* = 10), the VIP+ interneurons (Figure 5B, amplitude_pre_ = 6.5 ± 1.2, amplitude_post_ = 2.5 ± 1.1, *p* < 0.01, *n* = 10), and the PV+ interneurons with positive responses (Figure 5D, amplitude_pre_ = 3.6 ± 0.5, amplitude_post_ = −1.3 ± 1.7, *p* < 0.05, *n* = 9), indicating that the BF-induced increase in intracellular calcium of these neurons requires mAChR activation. For the PV neurons with negative responses, atropine reduced the response amplitude by 37.5% (Figure 5E, amplitude_pre_ = −3.2 ± 0.5, amplitude_post_ = −2.0 ± 0.3, *p* < 0.05, *n* = 26). Since the VIP+ interneurons have been shown to provide synaptic input to PV+ interneurons (Dávid et al., 2007), the reduced negative PV+ response may be caused by the reduced inhibition from VIP+ interneurons. In contrast to these significant effects on layer 2/3 neurons, atropine caused no significant change in the response amplitude of layer 1 inhibitory neurons (Figure 5C, amplitude_pre_ = 6.8 ± 1.8, amplitude_post_ = 6.9 ± 2.5, *p* = 0.45, *n* = 6).

**Figure 6:**
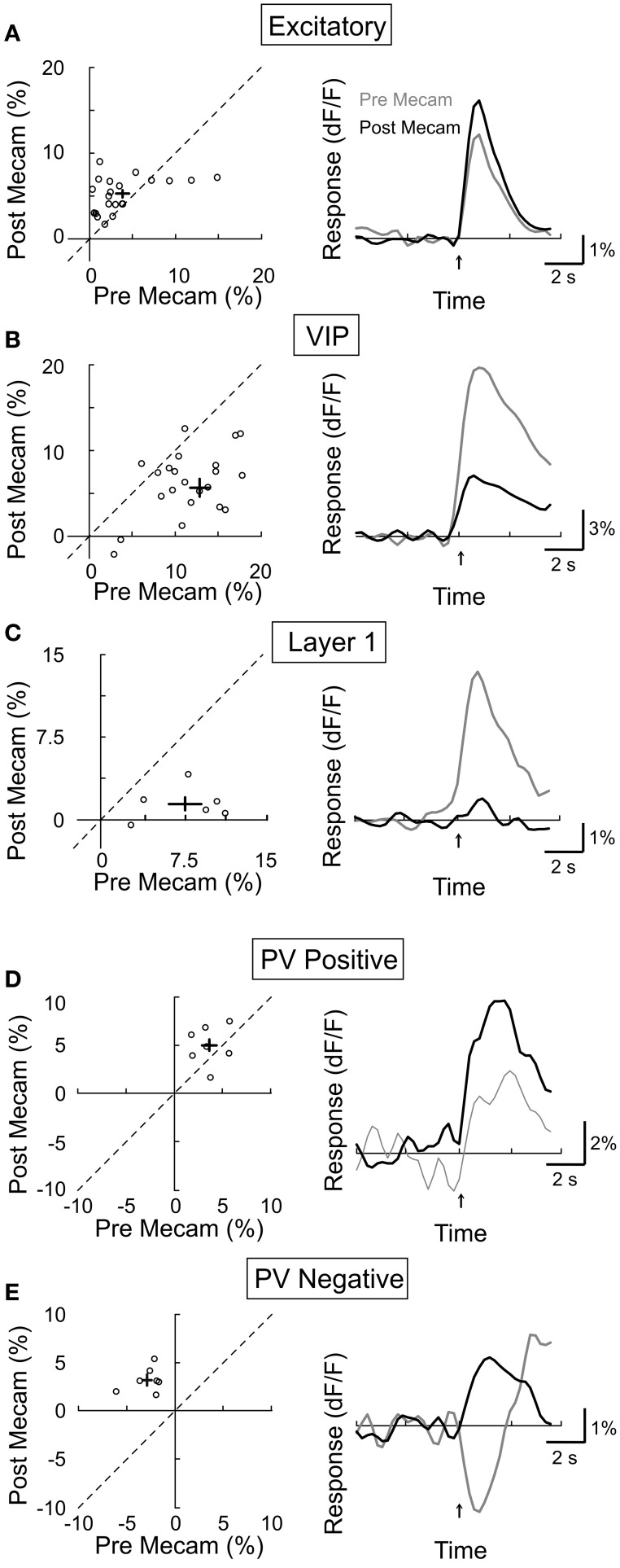
**Effect of mecamylamine on BF modulation of cortical neurons.** Left column, post-mecamylamine vs. pre-mecamylamine response amplitude (dF/F). Each symbol, one cell; error bar, ±SEM. Right column, average response from all responsive cells in each subtype; gray, pre-mecamylamine; black, post-mecamylamine. **(A)** Excitatory neurons, increased 27% post-mecamylamine (*p* = 0.05, *n* = 23). **(B)** VIP+, decreased by 57% (*p* < 0.0001, *n* = 25). **(C)** Layer 1, decreased by 80% (*p* < 0.01, *n* = 6). **(D)** PV+ with positive responses, increased by 39% (*p* = 0.17, *n* = 7). **(E)** PV+ with negative responses, decreased by 210% (*p* < 0.001, *n* = 7).

We next assessed the contribution of nAChRs by topically applying a specific nAChR antagonist mecamylamine (3 mM). We found that mecamylamine strongly reduced the response amplitude of both VIP+ (Figure 6B, amplitude_pre_ = 13.0 ± 1.1, amplitude_post_ = 5.6 ± 1.1, *p* < 0.0001, *n* = 25) and layer 1 interneurons (Figure 6C, amplitude_pre_ = 7.5 ± 1.5, amplitude_post_ = 1.5 ± 0.6, *p* < 0.01, *n* = 6), consistent with the finding that these neurons express AChRs (Porter et al., 1999; Christophe et al., 2002; Lee et al., 2010). Surprisingly, mecamylamine increased the response amplitude of both the CaMKIIα+ excitatory neurons (Figure 6A, amplitude_pre_ = 3.8 ± 0.8, amplitude_post_ = 5.2 ± 0.4, *p* = 0.05, *n* = 23) and the PV+ interneurons with positive responses (Figure 6D, amplitude_pre_ = 3.6 ± 0.6, amplitude_post_ = 5.0 ± 0.8, *p* = 0.17, *n* = 7), and it even converted negative PV+ responses to positive responses (Figure 6E, amplitude_pre_ = −2.9 ± 0.6, amplitude_post_ = 3.2 ± 0.5, *p* < 0.001, *n* = 7). Since the excitatory and PV+ neurons do not express nAChRs (Porter et al., 1999), and VIP+ and layer 1 interneurons are known to project to these neurons (Peters, 1990; Dávid et al., 2007; Wozny and Williams, 2011), the effects of mecamylamine suggest that VIP+ and/or layer 1 interneuron activation through nAChRs causes inhibition of the excitatory and PV+ neurons, leading to either negative responses or a reduction of the mAChR-mediated positive responses. Given that PV+ interneurons have been previously found to have relatively high spontaneous activity, it is likely that their below baseline, negative responses reflect a decrease in spiking activity (Gentet et al., 2010; Ma et al., 2010).

### Dependence on glutamatergic transmission

**Figure 7:**
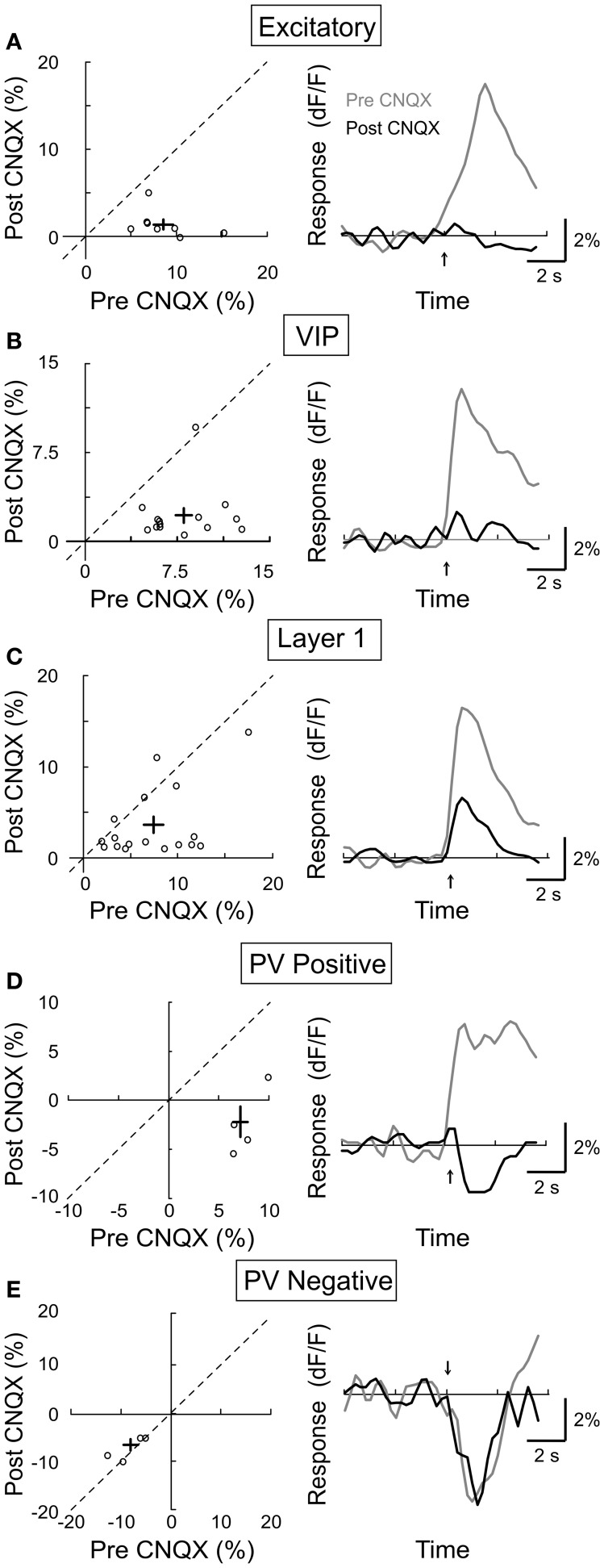
**Effect of CNQX on BF modulation of cortical neurons.** Left column, post-CNQX vs. pre-CNQX response amplitude (dF/F). Each symbol, one cell; error bar, ±SEM. Right column, average response from all responsive cells in each subtype; gray, pre-CNQX; black, post-CNQX. **(A)** Excitatory neurons, decreased by 85% post-CNQX (*p* < 0.001, *n* = 14). **(B)** VIP+, decreased by 73% (*p* < 0.001, *n* = 14). **(C)** Layer 1, decreased by 53% (*p* < 0.0005, *n* = 16). **(D)** PV+ with positive responses, decreased by 131% (*p* < 0.0005, *n* = 4). **(E)** PV+ with negative responses, no significant change (*p* = 0.6, *n* = 4).

In addition to the cholinergic input, the BF also provides glutamatergic projections to the cortex (Henny and Jones, 2008). Furthermore, cholinergic activation of the excitatory neurons (Figure 3A) may affect other cortical neurons through local glutamatergic interactions within the cortical circuit. We thus tested the role of glutamatergic synaptic transmission by topical application of the AMPA receptor antagonist CNQX (4 mM). We found that CNQX caused a large reduction in the response amplitude of excitatory neurons (Figure 7A, amplitude_pre_ = 8.6 ± 1.1, amplitude_post_ = 1.3 ± 0.6, *p* < 0.001, *n* = 14), VIP+ interneurons (Figure 7B, amplitude_pre_ = 8.1 ± 0.71.5, amplitude_post_ = 2.2 ± 0.6, *p* < 0.001, *n* = 14), and the PV+ neurons with positive responses (Figure 7D, amplitude_pre_ = 7.7 ± 0.8, amplitude_post_ = −2.4 ± 1.7, *p* < 0.0005, *n* = 4). This indicates that the NB-induced activation of these cells is partly mediated by glutamatergic interactions. Interestingly, the effects of CNQX on layer 1 neurons appeared to fall into two distinct groups: while it caused a strong reduction in the response amplitude of 8/16 neurons, it had little effect on the remaining layer 1 cells (Figure 7C, amplitude_pre_ = 7.6 ± 1.1, amplitude_post_ = 3.6 ± 1.0, *p* < 0.0005, *n* = 16). This is consistent with the recent finding that only a subset of layer 1 interneurons receive excitatory input from layer 2/3 (Wozny and Williams, 2011). For PV+ neurons with negative responses to NB stimulation, CNQX had no significant effect (Figure 7E, amplitude_pre_ = −4.1 ± 0.9, amplitude_post_ = −3.7 ± 0.6, *p* = 0.6, *n* = 4), consistent with the notion that the negative responses reflect inhibitory synaptic transmission. However, it is important to note that, along with excitatory synapses, potential sources of inhibition to PV+ interneurons were also suppressed by CNQX (i.e., VIP+ and layer 1 interneurons). Thus, the suppressive influences of CNQX on excitatory synapses and inhibitory interneurons may combine to produce no net effect on negative responding PV+ interneurons.

### Relationship with cortical desynchronization

**Figure 8:**
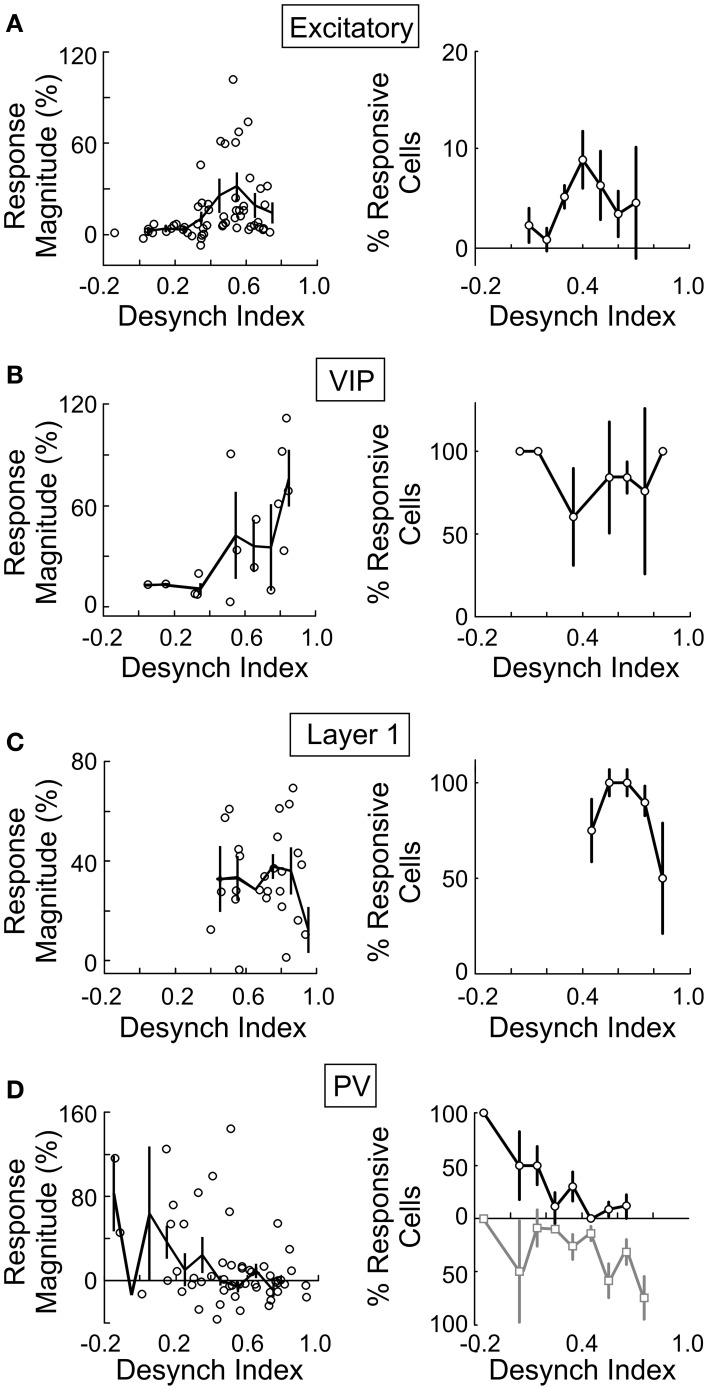
**BF modulation of individual neurons is correlated with cortical desynchronization.** For each cell type the response magnitude of significantly responsive cells (left column, each data point represents average from one experiment) and percentage of cells that were significantly responsive (right column) are plotted against the cortical desynchronization index (1 – EEG power Pre-Stim_1–10 Hz_/EEG power Post-Stim_1–10 Hz_), *n* = 145 experiments. **(A)** Excitatory cells. **(B)** VIP+ neurons. **(C)** Layer 1. **(D)** PV+ (black/gray, cells with positive/negative responses). Error bars, ± SEM.

**Figure 9:**
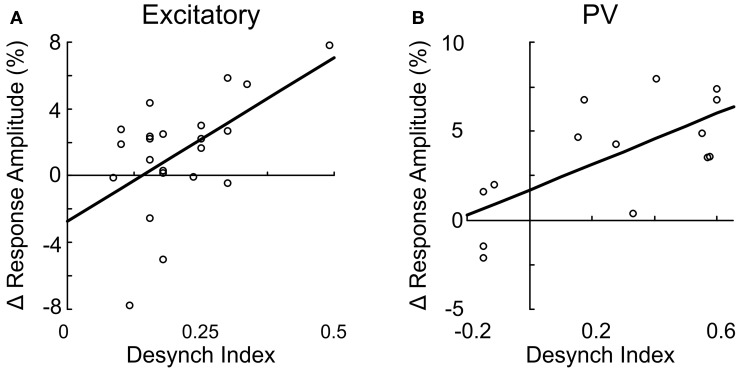
**Suppression of excitatory and PV+ neurons through nAChR is correlated with cortical desynchronization.** The change in response amplitude after mecamylamine application is plotted against the desynchronization index for excitatory neurons (**A**, *r* = 0.55, *p* < 0.01) and PV+ interneurons (**B**, *r* = 0.73, *p* < 0.01). Each data point represents one neuron. Line, linear fit.

Since both the EEG desynchronization (Metherate et al., 1992) and the calcium responses of individual neurons evoked by BF stimulation (Figures 5, 6) depend on AChRs, the magnitudes of these two effects are expected to be correlated. Indeed, we found that the response magnitude and the percentage of significantly responsive neurons were correlated with the desynchronization index of each experiment (Figure 8). However, there are important differences across cell types. The excitatory neurons began to respond at a desynchronization index of ~0.3, but the response appeared to decrease at desynchronization index >0.6 (Figure 8A). For VIP+ neurons, the response magnitude increased steeply with the desynchronization index, with a high threshold of ~0.5 (Figure 8B). Since mAChRs contribute positively to the responses of both excitatory and VIP+ neurons (Figures 5A,B), but nAChRs contribute negatively to the excitatory neuron responses (Figure 6A), these results suggest that cholinergic modulation is dominated by mAChR activation at low desynchronization levels, and the nAChR-mediated effects manifest primarily at high desynchronization levels. Consistent with this idea, we found that blocking nAChRs had little effect on the responses of excitatory neurons at low desynchronization levels, but a large effect at strong desynchronization (Figure 9A, *r* = 0.55, *p* < 0.01). For PV+ interneurons (Figure 8D), we found more positive responses at desynchronization index <0.4 and primarily negative responses at high desynchronization levels. As was the case for excitatory neurons, the sensitivity of PV+ interneurons to mecamylamine increased with the strength of cortical desynchronization (Figure 9B, *r* = 0.73, *p* < 0.01), indicating that the modulation of PV+ neurons is also mediated primarily by mAChRs (driving positive PV+ responses, Figure 5D) at low desynchronization and nAChRs (negative PV+ responses, Figure 6E) at high desynchronization.

## Discussion

**Figure 10:**
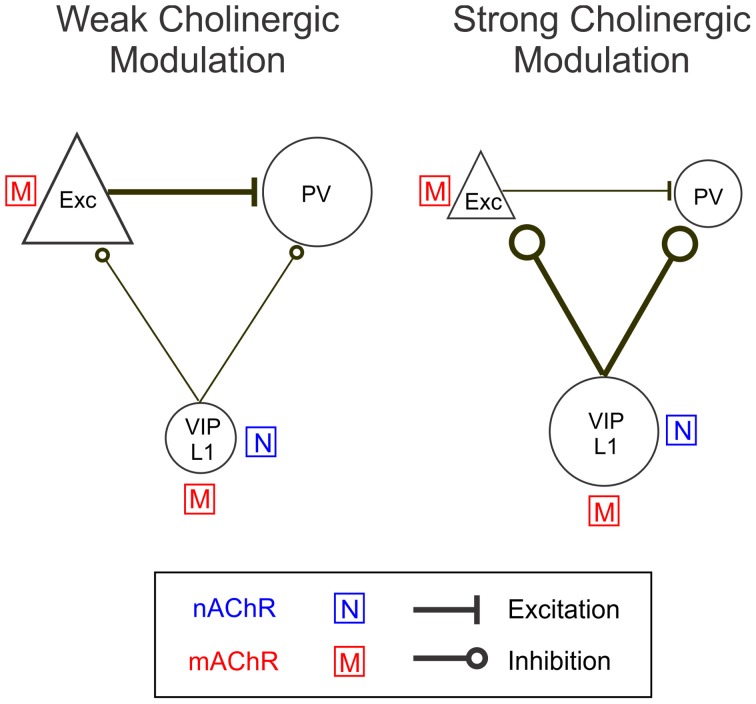
**A model circuit of cholinergic modulation of cortical neurons.** During weak cortical desynchronization muscarinic modulation is dominant, activating excitatory neurons directly and PV+ interneurons indirectly (through glutamatergic input from excitatory neurons). During strong cortical desynchronization nicotinic modulation becomes more pronounced, causing activation of VIP+ and layer 1 interneurons directly and a reduction in excitatory and PV+ neuron activity indirectly (through GABAergic inhibition from VIP+/layer 1 neurons). The sizes of the icons and thicknesses of connecting lines reflect the relative activity levels during weak and strong cortical desynchronization.

Our results show that BF activation modulates the activity of excitatory neurons and several subtypes of inhibitory interneurons. The pharmacology experiments and EEG correlation analysis can be summarized in the following model (Figure 10). With weak BF activation, indicated by low levels of desynchronization, excitatory neurons and PV+ inhibitory interneurons are strongly activated through mAChRs, whereas the VIP+ interneurons are only weakly activated. With stronger BF activation (strong EEG desynchronization), the nAChR-dependent responses of VIP+ interneurons increase sharply, while the responses of excitatory neurons are reduced and those of PV+ neurons switch from positive to negative. Thus, an increased activation of BF inputs appears to be associated with an increase in the relative contribution of nAChRs, causing a shift in cortical activity from excitatory and PV+ neurons to VIP+ and layer 1 neurons. A recent study in cortical slices showed that optogenetic stimulation of cholinergic fibers originating from the BF caused nAChR-mediated excitation of layer 1 and non-fast spiking layer 2/3 interneurons as well as inhibition of both pyramidal and PV+ neurons (Arroyo et al., 2012). Our findings are consistent with this previous result and further reveal how the nAChR-dependent effect interacts with the mAChR-mediated modulation in an intact network *in vivo*.

The diverse responses of these cell types are likely mediated by both the AChRs expressed on each cell type and the synaptic interactions within the local circuit, including substantial contributions of glutamatergic synapses (Parikh et al., 2008). Cortical pyramidal neurons are known to be depolarized by ACh through the activation of mAChRs (McCormick, 1992). The positive responses we have observed with calcium imaging are likely to reflect spiking of the subset of pyramidal neurons with strong depolarizing responses to ACh. Previous *in vitro* studies have shown that rodent PV+ neurons are not depolarized by ACh or other mAChR agonists (Kawaguchi, 1997; Xiang et al., 1998; Gulledge et al., 2007). In our case, the mAChR-dependent activation of PV+ interneurons could be a secondary consequence to the activation of the glutamatergic neurons. Consistent with this notion, the positive responses of PV+ neurons were blocked completely by CNQX application. Unlike PV+ interneurons, VIP+ interneurons have been found to be responsive to mAChR agonists *in vitro* (Kawaguchi, 1997). Thus, the mAChR-dependent component of the VIP+ response could be mediated by both the intrinsic mAChRs and glutamatergic input from nearby pyramidal neurons. The VIP+ and layer 1 interneurons are known to express nAChRs (Porter et al., 1999; Christophe et al., 2002), which explains the large reduction of their responses by mecamylamine application. These interneurons are also known to innervate pyramidal and non-pyramidal neurons (Peters, 1990; Dávid et al., 2007; Wozny and Williams, 2011). Given that ionotropic nAChRs directly depolarize neurons, their negative contributions to excitatory and PV+ neurons are most likely mediated by GABAergic inhibition from the VIP+ and layer 1 interneurons. PV+ interneurons are known to have relatively high spontaneous firing rates (Gentet et al., 2010; Ma et al., 2010). Thus, their below baseline, negative responses likely reflect decreased spiking activity.

Of course, in addition to the small set of synaptic interactions summarized in Figure 10, other pathways may also contribute to the observed effects. Cholinergic modulation of deeper layers likely influences activity in layers 1 and 2/3. For example, nAChRs increase the gain of thalamocortical synapses in layer 4 and activate supragranular projecting, low-threshold spiking interneurons in layer 5 (Xiang et al., 1998; Disney et al., 2007). Activation of presynaptic muscarinic receptors suppresses the release of both glutamate and GABA from cortical–cortical synapses (Kimura and Baughman, 1997; Hasselmo and Sarter, 2011), which could contribute to the observed effects of atropine (Figure 5). In addition to cholinergic input, glutamatergic axons in fact make up a significant percentage of the BF input to the cortex (~15%, similar to the percentage of ACh fibers) (Henny and Jones, 2008), which could explain at least in part the reduction of BF-induced positive responses after CNQX application (Figure 7). Cholinergic modulation and the activation of cortical glutamate receptors are also strongly interdependent. Blocking cortical glutamatergic synapses significantly reduces the amplitude of cholinergic transients (Parikh et al., 2008), and presynaptic nAChRs can trigger the activation of cortical glutamatergic synapses (McGehee et al., 1995; Gray et al., 1996; Gioanni et al., 1999). Since the calcium transients observed in our experiments primarily reflects spiking activity of cortical neurons, the effect of CNQX may also be caused by a general hyperpolarization of cortical membrane potentials, which decreases the probability of neuronal spiking induced by cholinergic input. Finally, the mechanisms of cholinergic modulation found in infragranular cortex may differ from those present in layers 1 and 2/3. For example, layer 5 fast-spiking interneurons, commonly identified as PV+, are suppressed by mAChRs and are not modulated by nAChRs (Xiang et al., 1998). Such differences in cholinergic modulation may reflect the different roles of these layers in cortical processing.

The switch from PV+ to VIP+/layer 1 GABAergic inhibition induced by BF stimulation may have important consequences on neuronal processing in the neocortex. On a basic level it may modulate the flow of sensory information while maintaining excitation/inhibition balance (Wehr and Zador, 2003). Specifically, nAChRs decrease the inhibitory influence of PV+ interneurons onto excitatory cells while increasing the inhibition from VIP+ interneurons. This may be one mechanism by which cholinergic modulation of the cortex enhances feedforward, thalamic input and sensory processing while suppressing non-sensory related recurrent activity (Kimura et al., 1999; Hsieh et al., 2000; Disney et al., 2007; Parikh et al., 2007; Goard and Dan, 2009). The PV+ interneurons are strongly driven by sensory input and regulate excitatory neuronal activity through feedforward inhibition (Porter et al., 2001; Beierlein et al., 2003; Cruikshank et al., 2010; Ma et al., 2010). Nicotinic suppression of PV+ inhibition could therefore increase the gain of excitatory neurons during sensory processing, complementing the effect of nAChRs on layer 4 thalamocortical synapses (Disney et al., 2007; Kawai et al., 2007; Parikh et al., 2008). While many of the properties of VIP+ interneurons have not been fully characterized, these neurons provide more dendritic inhibition to excitatory neurons and are more weakly driven by sensory stimulation than PV+ interneurons (Peters, 1990; Kerlin et al., 2010). The suppression of PV+ interneurons by nicotinic modulation has also been observed during fear conditioning (Letzkus et al., 2011), and it is thought to enhance behaviorally relevant sensory input. In addition, PV+ neurons are shown to be less active during alert than quiescent states (Gentet et al., 2010), which could also be related to the higher level of ACh during alert states. The VIP+ interneurons have been shown to be active during periods of increased sensory stimulation (Cauli et al., 2004) and may play a direct role in regulating cerebral blood flow (Cauli et al., 2004; Lecrux et al., 2011) to support the increased metabolic needs during heightened neural processing.

Similar to the cholinergic system, other neuromodulators such as dopamine, serotonin, and noradrenaline also promote wakeful brain states and alert cognitive processing, and they selectively target distinct populations of cortical inhibitory neurons as well (Kawaguchi and Shindou, 1998; Bacci et al., 2005; Glausier et al., 2009; Lee et al., 2010). The cholinergic system directly interacts with these neuromodulatory systems (e.g., activation of presynaptic nAChRs in the neocortex can increase the release of dopamine and noradrenaline from the subcortical fibers) (Role and Berg, 1996; Cao et al., 2005; Shearman et al., 2005). Interestingly, nAChR-sensitive interneurons, including layer 1 and VIP+ neurons, belong to a large, developmentally distinct category of GABAergic neurons that also expresses the excitatory ionotropic serotonin receptor 5HT3aR (Férézou et al., 2002; Lee et al., 2010). Furthermore, these neurons are suppressed by mu opioid agonists (Férézou et al., 2007), which are known to induce EEG synchronization and cortical inactivation(Young and Khazan, 1984; Férézou et al., 2007). Thus, the nAChR-sensitive cortical interneurons are ideally suited for integrating multiple neuromodulatory inputs for the dynamic regulation of brain state and cortical processing.

### Conflict of interest statement

The authors declare that the research was conducted in the absence of any commercial or financial relationships that could be construed as a potential conflict of interest.
